# Books and Authors

**Published:** 1911-07

**Authors:** 


**A Practical Dictionary.** By **Thomas Lathrop Stedman,** A.M., M.D., Editor of "Twentieth Century Practice of Medicine"; Editor of the "Medical Record." Illustrated. New York: William Wood & Co. 1911. (Limp leather. thumb indexed, $5.00.)

That improvement may be made in so technical a book as a medical dictionary is shown by the excellence of this publication which has just been sent out by William Wood & Company. There are few dictionaries which are not error-ridden and it has been the effort of the publishers to produce a book which shall be absolutely modern in its contents and as free of error as possible. To that end they have had this work prepared by a man of scholarly attainments and long editorial experience.

Stedman's Dictionary includes the following features, some of them entirely novel: It gives the derivation of all words (when known) from the Anglo-Saxon as well as Latin and Greek. Pronunciation is indicated whenever it is not self-evident, and accent marked in all the main titles and in most of the subtitles; tables of all the numerous, anatomical structures, such as arteries, muscles, nerves, sulci, convolutions, foramina, fossae, and pharmaceutical preparations, such as acids, tinctures, and the like. These are not given in tabular form across the page so as to confuse one in searching for words, but are run along in columns in alphabetical order, distinguished by different type. Other tables, such as of the elements with their symbols and atomic weights, thermometer scales, weights and measures, and the like, are placed in the appendix, with reference thereto under the catch-word in the body of the dictionary, thus not interfering with one's convenience in looking for words.

Besides the regular medical terms this dictionary includes: dental terms; chemical terms; veterinary terms; botanical terms; insurance terms; homeopathic terms; eclectic terms; biographical data (nationality, date of birth and death with the eponymic terms,) all the preparations of the United States and British pharmacopeias and national formulary. Chemical symbols are given with the names of acids, salts, and the like, and also entered as main titles referring back to the full name. It also includes mineral springs, giving pronunciation of place (if foreign), country, character of the water, and indications for its use; Thesaurus, that is, defining back from the English or popular terms to the scientific. Words which should begin with a capital are so printed. The spelling of U.S.P. is used.

**A Handbook of Practical Treatment.** In three volumes. By 79 eminent specialists. Edited by **John H. Musser,** M.D. Professor Clinical Medicine, University of Pennsylvania; and A. O. J. Kelly, M.D., Assistant Professor of Medicine, University of Pennsylvania. Volume II. Octavo of 865 pages. Illustrated. Philadelphia and London: W. B. Saunders Company. 1911. (Per volume: Cloth, $6.00 net; half morocco, $7.50 net).

There is little of criticism that one may find in this the second volume of an excellent work on treatment. It bears all the careful preparation which characterised the first volume. It is an excellent work in every respect and its value cannot be overestimated.

Some of the special features are of exceptional interest notably the articles on yellow and dengue fevers by James Carroll. Cardiovascular conditions are written of by Sir Clifford Allbutt, and John M. T. Finney writes of the surgery of the heart and the surgical complications of typhoid fever. J. William White and Alfred C. Wood contribute the portion devoted to syphilis and leave no phase of the disease or its treatment undiscussed. Their position regarding the use of salvarsan is unassailable. Gonococcic infections by Edward Martin are not given the prominence or space that the importance of the diseases demand, but in generally reviewing the subject Martin has left little to be said.

